# Conspecific interactions predict social transmission of fear in female rats

**DOI:** 10.1038/s41598-024-58258-6

**Published:** 2024-04-02

**Authors:** Sydney Seese, Carolyn E. Tinsley, Grace Wulffraat, J. Gregory Hixon, Marie-H. Monfils

**Affiliations:** 1 Department of Psychology, University of Texas at Austin, 108 E. Dean Keeton Stop A8000, Austin, TX 78712-1043 USA; 2 Department of Neurology, Oregon Health and Science University, Portland, OR USA

**Keywords:** Social neuroscience, Emotion

## Abstract

Social transmission of fear occurs in a subset of individuals, where an Observer displays a fear response to a previously neutral stimulus after witnessing or interacting with a conspecific Demonstrator during memory retrieval. The conditions under which fear can be acquired socially in rats have received attention in recent years, and suggest that social factors modulate social transmission of information. We previously found that one such factor, social rank, impacts fear conditioning by proxy in male rats. Here, we aimed to investigate whether social roles as determined by nape contacts in females, might also have an influence on social transmission of fear. In-line with previous findings in males, we found that social interactions in the home cage can provide insight into the social relationship between female rats and that these relationships predict the degree of fear acquired by-proxy. These results suggest that play behavior affects the social transfer/transmission of information in female rats.

## Introduction

Learning from a conspecific’s aversive experience is an important means of acquiring information about potential threats while avoiding the personal risk of direct exposure. The ability to socially learn about threatening stimuli has been demonstrated in a number of species^1–6^ and the degree of learning can be modulated by the nature of the social relationship between observer (the social learner) and demonstrator (the direct learner)^7,8^. These relational factors can include familiarity^4,6,8–12^, kinship^4,8,9,13,14^, mating pairs^14,15^, and dominance status^4,16,17^. Dominance relationships as measured by play behavior have been well-established in male rodents^18^ and while play behaviors have also been observed in female rodents and related to dominance-like relationships^19–22^, their impact on social transmission of information has not been examined.

Unlike investigations in mice where dominance relationships are generally characterized by fighting^23–27^, triadic dominance relationships in rats can be characterized by their play behavior^18,22,58^. Similar to humans, rats engage in play from a young age, and doing so is thought to be essential for relationship management^28^. Play behaviors arise in prepubescence and reach their peak at the end of adolescence for both males and females^29^. While the frequency of play decreases with age, it continues into adulthood in males^29^ and is posited to be an essential means of conflict reduction and relationship maintenance^28,30–34^.

In males, the dominant rat (D) is characterized as the play partner that receives the most nape contacts (HNC). The male that receives the fewest nape contacts in the group (LNC), also the least likely to playfully engage, is classified as the second subordinate (S2). The rat that receives the most nape contacts from the dominant, but the one that is the second most recipient of nape contact (middle) (MNC) is the most subordinate rat (S1)^4,16–18^. How each male responds to those nape contacts also factors in determining his assigned role. Dominant males are most likely to respond to nape contacts with counterattacks, S1 males are most likely to rotate, and S2 males are the most likely to evade^16,18^. While there have been a few investigations into dominance hierarchies between females (see Table 1) there has been no consistent classification of female play behavior (i.e. nape contacts or response to nape contacts) as an indicator of dominance hierarchy.

**Table 1:** Past dominance investigations in female rats.

Species	Environment	Dominance test	References
Wild-type Norway	Cages within a laboratory environment	Resources monopolization of high value food item (banana)	^35^
Wild-type Norway	Cages within a laboratory environment	Resources monopolization of high value food item (banana)	^36^
Long Evans	Cages within a laboratory environment	Play behavior (pinning): resource access following deprivation (water)	^22^
Long Evans	Seminatural (colony cages connected by tubing system)	Female-female mounting behavior; agonistic behavior (lunging, biting, chasing, fleeing, rolling over)	^37^
Wild rats; Sprague Dawley	Seminatural (burrow system in laboratory environment)	Passing behavior; agonistic behavior (fights, shoves, boxes, chases, bites); resource access (food, water)	^20^
Wistar rats; Long-Evans	Seminatural (colony box in laboratory environment); cages in laboratory environment	Resident-intruder paradigm (observed rat is the resident); resource access following deprivation (food, water)	^38^
Sprague Dawley	Natural (outdoor pen)	Play behavior (pinning); agonistic behavior (biting posturing, chasing)	^19^
Long Evans	Cages within a laboratory environment	Social behavior (pinning, pouncing, boxing, mounting); resource access following deprivation (water)	^22^
*Cacna1c*^+*/−*^ rats; Wild-type *Cacna1c*^+*/*+^ rats	Large unfamiliar arena and cages within a laboratory environment	Social behavior (sniffing, following, social grooming, crawling over/under); aggressive behavior (piloerection, attack, tail wiggling, boxing); tube test of social dominance	^39^
Norway rat	Seminatural (burrow system in laboratory environment)	Social behavior (sniffing, following, social grooming); aggressive behavior (piloerection, chasing, mounting, attack, tail wiggling, boxing)	^40^

A comprehensive table of previous articles that have employed methods of dominance assessment in female rats. Importantly, these investigations are limited to successful investigations of female dominance (where a stable hierarchy was observed). Keywords for literary search: “Dominance”, “Female”, “Rat”. Date of latest search: March 2^nd^, 2023. Search engine: Google Scholar.

Previous work in mice and rats demonstrates that social rank has a significant effect on the ability to learn information from other conspecifics^4,16^. Kavaliers et al.^4^ previously showed that subordinate mice learn defensive and avoidance behaviors more effectively from dominant demonstrators than dominant observers did from subordinate demonstrators. Consistent with those findings, we previously found that subordinate male rats socially acquired fear more effectively from dominant demonstrators than dominant males did from subordinates^16^. Social acquisition of conditioned fear was investigated using a fear conditioning by proxy paradigm in which fear responding to a cue is socially transmitted to an observer rat who is freely interacting with a fearful conspecific demonstrator who was directly fear conditioned the previous day^41^. With this paradigm, we have consistently found subsets of pairs that acquire fear by proxy and subsets of pairs that do not, in both females and males^7,9,16,41^. Despite the fact that females demonstrate socially conditioned freezing in the fear conditioning by proxy paradigm, no one has this far assessed whether social role modulates fear conditioning by proxy in females.

In this paper, we aimed to examine how play behavior roles in female rats influence the expression of socially learned fear. We assessed play behavior of females using nape contacts, as this is a measure employed to assess social rank in males^18^. Based on previous work in male rats, we hypothesized that fear conditioning by proxy in females would be influenced by the social roles they display in play behavior, and would be dependent on the number of nape contacts received by the observer. This hypothesis is consistent with our finding that male rats demonstrate less fear by proxy when learning from a subordinate than if they are learning fear from a more dominant male^16^. We report socially and non-socially acquired fear expression (freezing) in virgin female rats and compare social play interactions sampled from male and female triads (male freezing comparison data and play data were previously published in Jones and Monfils^16^).

## Methods

### Experimental overview

In order to determine how social role influences social transmission of information, we first determined their social roles (details below) during play behavior within triad-housed female littermates^16,18^. Estrus cycles were tracked with daily vaginal swabbing prior to the start of fear conditioning (details below). After role determination, the rats were run through our fear conditioning by proxy procedure, as previously described^16,41^. Briefly, one member of the triad was fear conditioned to a tone conditioned stimulus (CS) paired with a shock (unconditioned stimulus, US). The next day, the fear conditioned rat (demonstrator, or FC) was returned to the fear conditioning chamber with a naïve cagemate (observer, or FCbP), where they were exposed to 3 CS presentations (details below). The next day, the demonstrator, observer, and a naïve cagemate were individually exposed to the tone CS and their freezing was assessed offline^16^.

### Subjects

Subjects were adult female Sprague Dawley rats bred at the University of Texas at Austin, from male breeders obtained from Harlan (now Envigo) and female breeders obtained from Charles River Laboratories. 10 breeder pairs contributed to the subjects used in these experiments and litters were counterbalanced across groups as best as possible depending on litter size and sex ratios. Rats were housed in temperature- and humidity-controlled rooms on a 12 h:12 h light:dark cycle, with lights on at 0700 h. Pregnant dams were checked two times a day for the presence of litters and the discovery of pups was marked as postnatal day 0 (P0). Litters were weaned at P21 into same sex triads with littermates. These studies were conducted in tandem with a similar experiment on the male littermates^16^. Males and females were housed in the same colony room and food (Purina rat chow) and water were provided ad libitum. Male data (n = 154) from Jones and Monfils^16^ is used for the sake of comparison in play behavior analysis and fear conditioning by proxy analysis.

### Play observations

Play behavior was recorded 3 weeks prior to the start of fear conditioning according to the methods of Pellis and Pellis^18^ when rats were approximately 100 days old. Each triad was separated and single housed for 24 h. After 24 h, triads were reunited at the start of their dark cycle in a chamber lined with bedding and recorded under red light illumination for 10 min. This was repeated for a total of 3 sessions.

### Nape contact and response scoring

Videos of play observations were watched at reduced speed and each rat in the triad was scored (with r = 0.97 inter-rater correlation) for nape contacts initiated as well as initial response to each nape contact received. Behaviors were summed for the three total sessions and in each cage, and social roles for each rat were assigned based on number of nape contacts received. Specifically, a nape contact was recorded when a one of the triad members brought their snout within 1 cm of the nape of another triad member. Snout distance from all other body targets was not recorded during video scoring. The member of the triad that received the most nape contacts was classified as Highest Nape Contacted (HNC). The rat that received the second most nape contacts was classified as Middlemost Nape Contacted (MNC). The final member of the triad was classified as Least Nape Contacted (LNC). In addition to nape contact initiation and receipt being recorded for each triad member, their response to the nape contact, if a response was observed, was scored as either evasion (target rat flees or pulls nape away from attacker), counterattack (target rat turns to face attacker and launches an attack of his own; boxing was included in this if it was in response to a nape contact), or rotation (counted full rotation to supine position, and half rotation with feet planted)^16^. We chose assignments of HNC, MNC, and LNC because number of nape contacts received and initiated are also used in males to predict social rank, detailed in Jones and Monfils^16^. By targeting nape contacts as a measure of role in play behavior, we remove the risk of classification based on more male-typical measures, such as frequency of counterattack. Play behavior analysis was completed in 11 triads (n = 33 female rats), see Supplementary Materials and Supplementary Figure S1 for information on excluded groups.

### Fear conditioning by proxy

**Figure 1:**
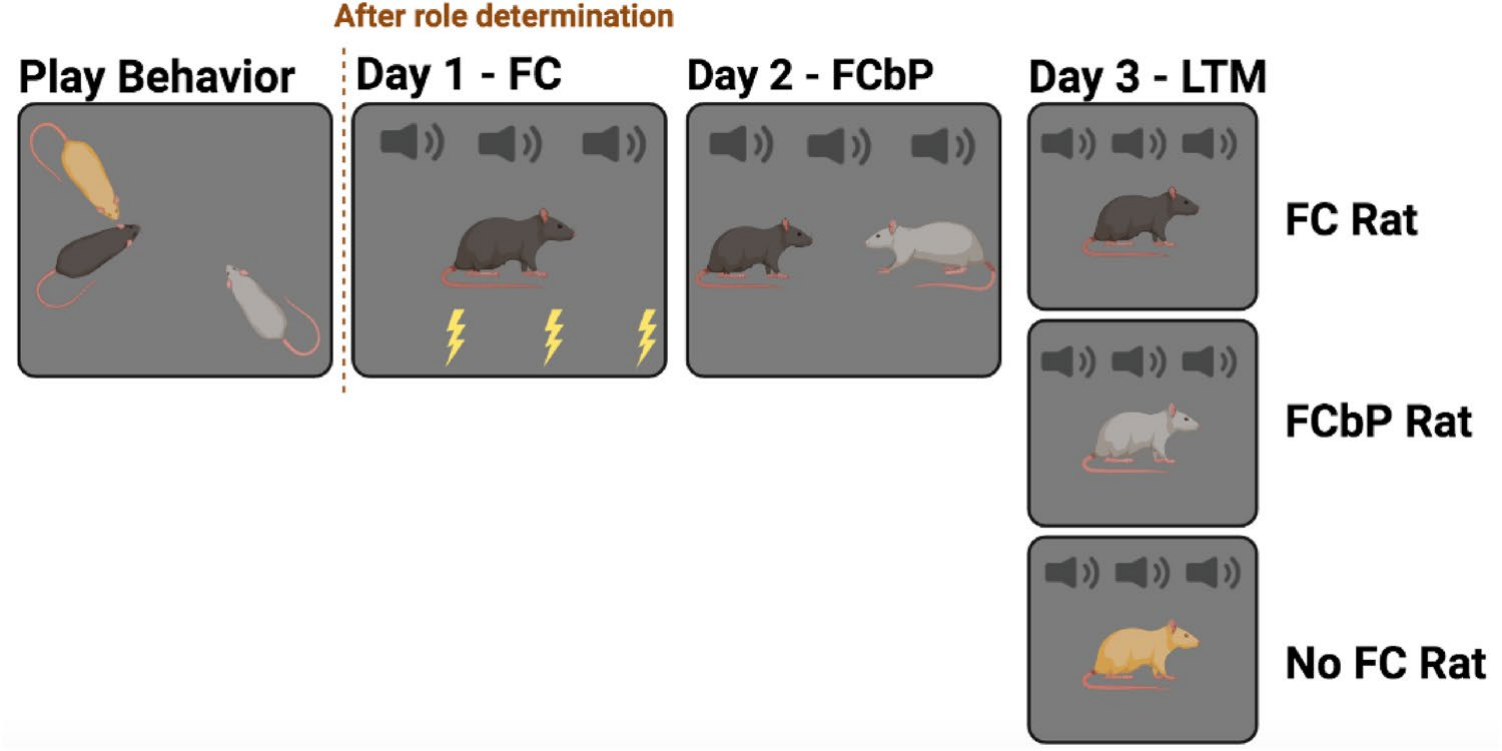
Experimental Design. Play Behavior: Play behavior was assessed 3 weeks before the start of fear conditioning and involved isolating each member of the triad for 24 h and then bringing all members of the triad together for a 10-min play observation. This process was completed three times. Prior to fear conditioning, 2 full estrus phases were sampled from each rat, using vaginal cytology. Fear Conditioning (FC): After social roles were determined, one member of the triad (either the presumed dominant or highest nape contacted (HNC) or the presumed second highest in the social rank, least nape contacted (LNC)) was fear conditioned to a tone (5 kHz, 80 db, 20 s in length) which co-terminated in a 0.7 mA, 500 ms footshock. This tone shock pairing was repeated 3 times in total. Fear Conditioning by Proxy (FCbP): 24 h after fear conditioning, the fear conditioned rat and a naive cagemate were returned to the FC chambers and allowed to freely interact while being exposed to the same tone 3 times, without a shock pairing. Long Term Memory Test (LTM): 24 h after Fear Conditioning by Proxy, all members of the original triad (fear conditioned demonstrator, fear conditioned by proxy observer, and rat with no previous fear conditioning) were placed in the chambers alone and exposed to the same 3 tones. Freezing behavior was recorded during all FC, FCbP, and LTM tests.

Social fear learning was assessed using the fear conditioning by proxy (FCbP) paradigm previously described^9,16,41^ when female rats were approximately 120 days old. FCbP is a three-day paradigm (see Fig. 1). On day 1, one rat from each triad was fear conditioned directly to three tones (5 kHz, 80 dB, 20 s duration), each coterminating with a footshock (0.7 mA, 500 ms duration, variable inter-trial interval (ITI): 180 s). The conditioned stimulus (CS) was a tone (5 kHz, 80 dB) 20 s in duration, and the unconditioned stimulus (US) was a 0.7 mA foot-shock 500 ms in duration. After fear conditioning, the fear conditioned rat (FC) was returned to the home cage. On day 2, the fear conditioned rat was returned to the fear conditioning chamber accompanied by a previously naïve cagemate (two rats present in the chamber) and the CS tone was played 3 times (20 s duration each time, variable ITI). Both FC and Fear Conditioned by Proxy (FCbP) rats were returned to the home cage. On day 3, all three rats from the triad were tested alone for fear response to the tone (3 tone presentations in fear conditioning context, 20 s duration for each tone presentation, variable ITI—long term memory test). This form of testing controls for potential effects of social transfer of information in the home cage by including the third, previously naïve cagemate. Cages were assigned to one of two groups based their play behavior-derived social status. In one group, the HNC acted as Demonstrator, and the LNC acted as Observer. In the other group, the LNC was Demonstrator, and the HNC was Observer. Fear conditioning analysis was completed in 11 triads (n = 33 female rats), see Supplementary Materials and Supplementary Figure S1 for information on excluded groups.

In Jones and Monfils^16^, we examined all permutations of FCbP in our triads. As such, Dominant, Subordinate 1 and Subordinate 2 rats (equivalent to HNC, MNC, and LNC here) were all Fear Conditioned directly, Fear Conditioned by Proxy, and not Fear Conditioned. Our results yielded group differences between all ranks, but the biggest effect was observed between the Dominant and the Subordinate 2 rats (roughly equivalent to the HNC and LNC here). For the present study, given the number of rats we had available in our colony for our experiment at the time, we did not have the power to examine all permutations. We thus chose to prioritize examining the HNC and LNC. We plan to investigate the role of the MNC observers and demonstrators in female rats in future experiments, in an effort to further understand potential differences or similarities in the strength of play behavior predictability to describe social learning between male and female rats.

### Behavioral scoring of fear conditioning by proxy

Videos were scored by an investigator blind to experimental condition and dominance status. Total time spent freezing during each tone presentation was assessed with a digital stopwatch. Freezing was defined as the absence of movement excluding respiration and the freezing for each of the three tones was averaged for the full session and expressed as a percentage (60 s total were scored for each video). In addition to freezing, social interactions that occurred between the observer and demonstrator within the testing chamber were assessed both during the presentation of the tone and in the 20 s immediately following termination of the tone (see^9^ and^41^ for full details regarding social interactions).

### Estrous cycle determination

Vaginal smears were collected daily using normal saline and a micropipette for 11 days prior to the start of fear conditioning by proxy and approximately 2 weeks after the completion of social interaction observations. Estrous cycle was determined by examining the cytology of fresh samples under a microscope at 10 × and coded as either proestrus (presence of nucleated cells), estrus (predominately cornified cells), or diestrus (including both diestrus 1 and diestrus 2, predominately leukocytic cells)^9,42,43^. If metestrus was observed, it was coded as diestrus. Vaginal smears were collected 3 h after lights-on each day of smearing and approximately 2–3 h prior to behavioral testing.

### Statistical analysis

In order to evaluate the strength of behavioral predictors of social role, we analyzed potential predictors using a multinomial regression from the nnet library^44^ in R^45^. Visualization of the strength of each predictor in the results section was completed using the effects library^46–48^. To assess the count of nape contacts initiated and received in males compared to females, which were over dispersed, we ran a generalized linear model using the negative binomial distribution by utilizing the glm.nb function from the AER library^49^ and MASS library^44^ in R. For dichotomous outcomes measures—such as probability for each rat to respond to each received nape contact with either evasion, counterattack, or rotation—the data were input into a generalized linear mixed effect model, using the lme4 package in R^50^. For continuous outcome measures, such as percent of time spent freezing in LTM, we used the lm function from base R^45^. It should be noted, influence of dominance status on the freezing behavior of the demonstrators and observers in males was restricted to the female design of only HNC and LNC observers and demonstrators, with the non-fear conditioned group being the MNC. When multiple candidate predictors were being assessed, we used the step function from the MASS package^44^ with direction set to “both” which uses the AIC criterion to arrive at a full model that includes only terms that meaningfully contribute to the prediction of the model. To assess the statistical importance of each term in the model, the anova function from base R^45^ was used to compare the full model to the reduced model that omitted the predictor of interest being evaluated using a chi-squared likelihood ratio test. In the case of models with a single focal predictor, the anova function was used to compare both the model with the single predictor to the null model with no predictors, again using a chi-squared likelihood ratio test.

See supplementary materials for statistical analysis code and equations (predictors and outcome).

All parts of this experiment were conducted in compliance with the National Institutes of Health Guide for the Care and Use of Experimental Animals and were approved for use by The University of Texas at Austin Animal Care and Use Committee. The studies reported in this manuscript were conducted in accordance with ARRIVE guidelines.

## Results

The male play and freezing behavior presented here were previously published in Jones and Monfils^16^. These data were collected at the same time as the female data presented here, which are novel.

### Sex differences in play behavior

**Figure 2:**
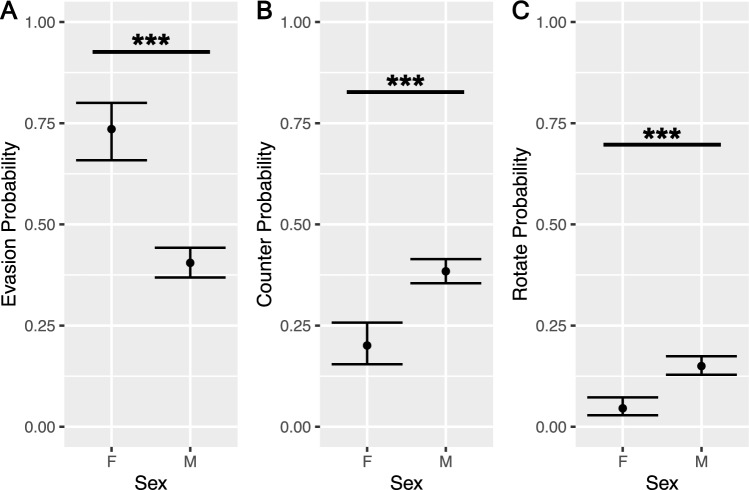
Social interactions in triads of male and female rats. Social interactions in triads of male and female rats were observed for 10 min during the dark cycle after a 24-h period of social isolation. Three sessions of play were recorded for nape contacts initiated as well as initial response to each nape contact received: evasion, rotation, or standing counter attack. Black points and error bars correspond to best model predictions for mean and confidence intervals. A) Females were more likely to evade following a nape contact from a related same-sex cagemate than males. B and C) Males were more likely to respond to nape contacts by engaging in a form of facing defense including either standing counter attacks or a full or partial rotation to supine. (* =  < 0.05, ** =  < 0.01, *** =  < 0.001) (Male data from: Jones and Monfils^16^).

A generalized linear model using the negative binomial distribution was run, and revealed that, in-line with the findings from previous studies^29,51–53^ that compared male and female rat play behavior, males received significantly more nape contacts (${\chi }^{2}\left(1\right)=13.83,p=2.01e-04, residual\, df=185$), and trended towards initiating more nape contacts (${\chi }^{2}\left(1\right)=3.17,p=0.075, residual\, df=185$), than females (see Supplementary Figure S6). Using generalized linear mixed effect models, the probability for a subject to demonstrate a particular response to a nape contact was calculated. We found that females were most likely to demonstrate an evasion response to a nape contact and were significantly more likely to do so than males (${\chi }^{2}\left(1\right)=44.30,p=2.82e-11, residual\, df=7299$). In contrast, we found that males were more likely to demonstrate a counterattack (${\chi }^{2}\left(1\right)=25.98,p=3.46e-07, residual\, df=7299$) or rotation to supine position after a nape contact than females (${\chi }^{2}\left(1\right)=24.47,p=7.53e-07, residual\, df=7299$) (see Fig. 2).

### Play behavior role influences response to nape contacts during play

**Figure 3:**
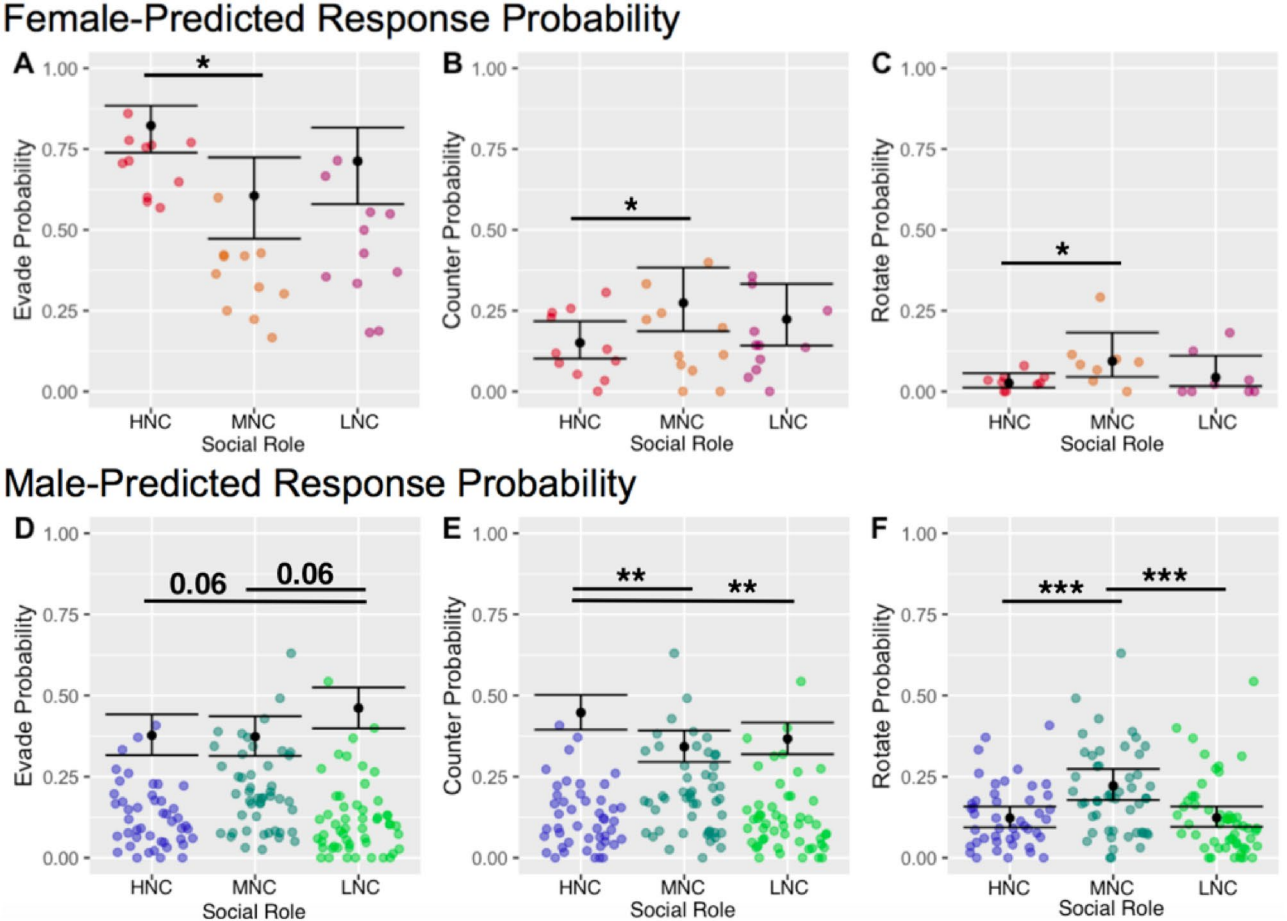
Male and female play behavior is influenced differently by social rank. (Top) In contrast to the findings in males (presented on the lower half of the figure), females that are assigned social role using nape contact show differences in play behaviors relative to males, such that HNC females are more likely than MNC females to respond to a received nape contact with evasion. Furthermore, MNC females are more likely than HNC females to demonstrate counterattack behavior to a nape contact. Rotation responses are similar to those observed in males, with MNC showing greater likelihood to rotate in response to a nape contact than HNC females. (Bottom) These play behaviors, analyzed in male data that were collected during a similar time frame as the female data but previously published in Jones and Monfils in 2016^16^, are comfortably within the previous literature for what is expected of male play behaviors. LNC males are the most likely to exhibit evasion in response to a nape contact, HNC males are the most likely to counterattack when receiving a nape contact, and MNC males are the most likely to rotate into a supine position after receiving a nape contact^16^.

Using generalized linear mixed effect models, the probability for a subject to demonstrate a particular response to a nape contact was calculated. Despite the fact that nape contacts are less frequent in females than males, play observation consistently showed that HNC females demonstrated unique play behavior from other female social roles. When each rat in the cage was assigned a social role according to the criteria described in the methods (see Nape contact and response scoring), generalized linear effect models with social status as the predictor and response type as the output revealed that the likelihood to respond to nape contacts with evasion was significantly greater in the HNC female compared to MNC (${\chi }^{2}\left(1\right)=7.39, \; p=0.0065, \; residual\, df=717$). This finding is unique from the males who demonstrate the greatest likelihood to evade when they are LNC (Compared to HNC: ${\chi }^{2}\left(1\right)=3.49,p=6.17e-02, residual\, df=4183$, Compared to MNC: ${\chi }^{2}\left(1\right)=3.33,\; p=6.80e-02, \; residual\, df=4162$). Countering in females is significantly more likely to occur in response to nape contact in MNC females than in HNC females (${\chi }^{2}\left(1\right)=3.85,\; p=0.050, \; residual\, df=717$). Again, this is different from males in which the HNC male is the most likely to counterattack when nape-contacted by a MNC or LNC cagemate (Compared to MNC: ${\chi }^{2}\left(1\right)=7.81,\; p=5.19e-03,\; residual\, df=4526$, Compared to LNC:${\chi }^{2}\left(1\right)=4.62,\; p=3.16e-03, \; residual\, df=4183$). Finally, female MNC (${\chi }^{2}\left(1\right)=6.29,\; p=0.012,\; residual\, df=717$) social ranks are significantly more likely to rotate in response to nape contact than HNC females. In males, this is somewhat consistent, with MNC males being the most likely to rotate (Compared to HNC: ${\chi }^{2}\left(1\right)=11.67,\; p=6.35e-04, \; residual\, df=4526$, Compared to LNC: ${\chi }^{2}\left(1\right)=12.05,\; p=5.19e-04, \; residual\, df=4162$), though LNC males are not significantly different in probability to rotate compared to HNC males (${\chi }^{2}\left(1\right)=7e-04,\; p=0.98, \; residual\, df=4183$) (see Fig. 3).

### Social play relationships within a cage predict social fear transmission in females

**Figure 4:**
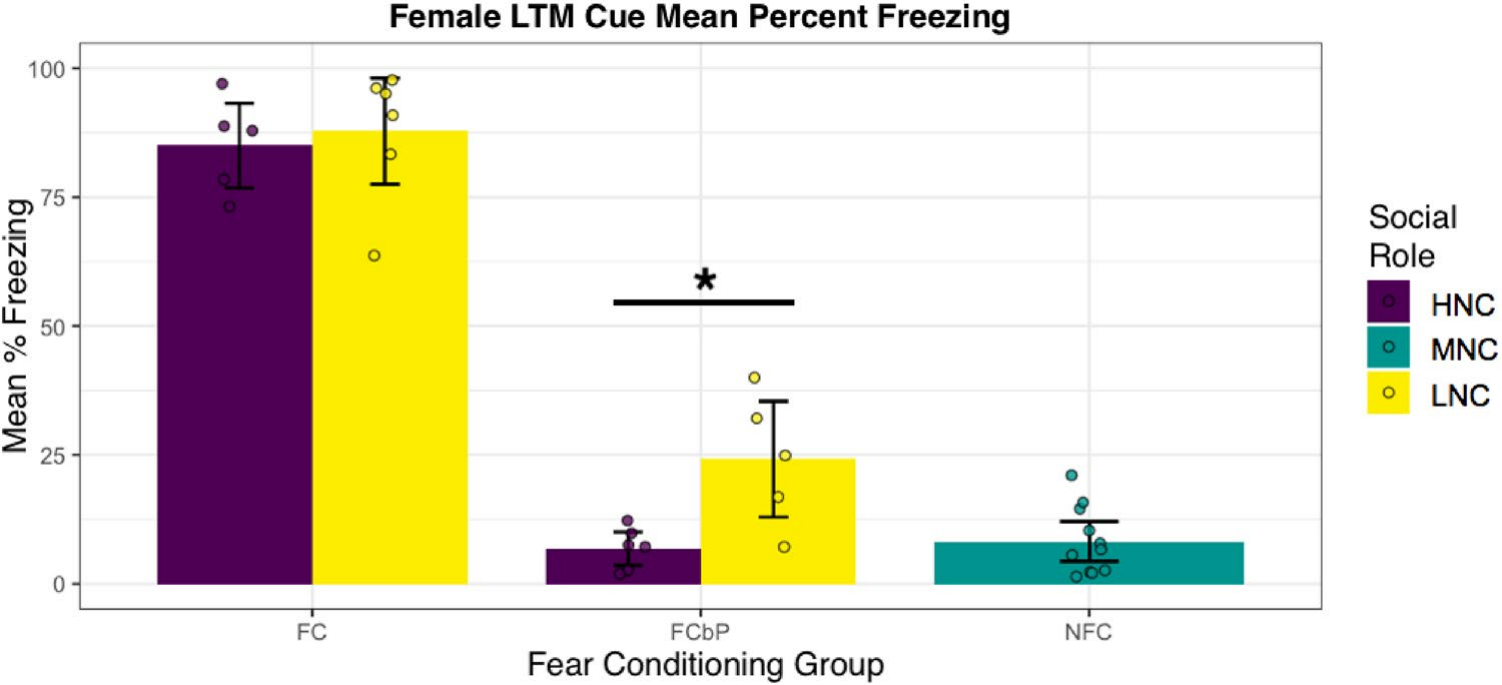
Mean percent freezing to conditioned stimulus at LTM after direct fear conditioning (FC and fear conditioning by proxy (FCbP). Mean percent freezing to conditioned stimulus at LTM is most affected by social role in FCbP. Rats froze more after observing and interacting with a HNC demonstrator within the cage than after observing and interacting with a LNC demonstrator. High Nape Contacted observers are presented above in purple, Low Nape Contacted observers are presented above in yellow. Non fear conditioned MNC females are presented in teal. The results in females and males are consistent with published male-only data from littermates that were collected in our lab during the same time period^16^. Mean percent freezing of each group throughout a 20 s cue is presented above. X-Axis acronym key: FC: Fear Conditioning, FCbP: Fear Conditioning by Proxy, NFC: No Fear Conditioning. Color key: HNC: High Nape Contact, MNC: Middle Nape Contact, LNC: Low Nape Contact.

A linear model was conducted and revealed that, consistent with prior work in males^16^, cued freezing behavior in the fear conditioning by proxy paradigm was determined by both the mode of fear exposure and the play behavior of the demonstrator in females. The analysis revealed that LNC observer females exhibited more freezing after observing the HNC demonstrator than HNC females who observing the LNC demonstrator ((Linear regression, main effect of FC group $F\left(\mathrm{1,93}\right)=248.69,\; p<2.2e-16$; main effect of demonstrator social status $F\left(\mathrm{1,93}\right)=3.04,\; p=0.084$; interaction $F\left(\mathrm{1,92}\right)=21.41,\; p=1.21e-05$); LNC freezing compared to HNC freezing: $F\left(\mathrm{1,26}\right)=15.29,p=5.9e-4$) (see Fig. 4). While significant differences between observer social ranks and freeing behavior were observed after FCbP, these differences are not the result of increased freezing behavior of the demonstrator during FCbP (see Supplementary Figure S3). For direct fear conditioning, there was no difference between the LNC fear conditioned subjects and the HNC fear conditioned subjects ($F\left(\mathrm{1,45}\right)=1.95,\; p=0.17$)). For further information about freezing behavior of demonstrators during direct fear conditioning and fear conditioning by proxy see Supplementary Figures S4 and S5.

There was a significant interaction of Sex and Fear Conditioning Role (F(1,217) = 8.15, *p* = 0.0047), such that male FCbP LNC rats froze more than Female FCbP LNC rats during Cue 2 in the Long Term Memory test (t(10.43) = 2.31, *p* = 0.043). (see Supplementary Figure S2).

### Female social role may be related to vaginal cell cytology

After running a multinomial regression, we found that frequency of proestrus, estrus, and diestrus did not significantly predict social role in females ($proestrus: {\chi }^{2}\left(2\right)=0.16,\; p=0.92, residual \; df=58; \; estrus: {\chi }^{2}\left(2\right)=2.43,\; p=0.30, residual \; df=58; \; diestrus: {\chi }^{2}\left(2\right)=1.61,\; p=0.45, \; residual \; df=58$). Despite this nonsignificant difference, we did find that HNC and LNC females trended towards more frequently experiencing at least two days of cornified epithelia cell type present in vaginal cellular environment compared to MNC females (ß = − 1.74, *p* = 0.15), with a moderately strong effect size (HNC vs. MNC: Cohen’s D = 0.66, LNC vs. MNC: Cohen’s D = 0.66). Greater cornified epithelia cell type in HNC and LNC females compared to MNC females suggests that HNC and LNC females may experience more frequent estrus than MNC females, perhaps also indicating more frequent periods of sexual receptivity. For visualization, see Supplementary Figures S7 and S8 (see^54^ for more information regarding vaginal cell environment).

## Discussion

In the current study, we sought to evaluate the influence of social behavior in triads of female rats on the social transmission of fear. We analyzed play behavior in triads of females as previously described in males^16^, and identified the rat that received the most nape contacts (HNC), the rat that received the most nape contacts from the HNC (MNC), and the rat that engaged the least and received the fewest nape contacts (LNC). In our female triads, the HNC rats showed more evasion than MNC females, and the MNC showed the most countering compared to HNC. Finally, female play behavior role significantly influenced fear conditioning by proxy, consistent with our previous findings in males. Higher nape contacted female observers froze less to conditioned fear demonstrated by a lower nape contacted female, and lower nape contacted female observers froze more to conditioned fear that was demonstrated by a higher nape contacted female. Based on these findings, we conclude that the relationships between female rats, as described by their play behavior, influence social transfer of information.

In male rats, the dominant rat receives the most nape contacts in the group (Dominant (D); High Nape Contact (HNC)). In each triad, the rat that receives the most nape contacts from the dominant is the most subordinate rat (Subordinate 1 (S1)). This rat also typically receives the second most nape contacts (Middle Nape Contact (MNC)). The third male typically receives the fewest nape contacts in the group, and is classified as the second subordinate (S2; Low Nape Contacts (LNC))^4,16–18^. In a typical male rat triadic hierarchy, the dominant males are most likely to respond to a nape contact with a counterattack, the S1 males are most likely to rotate, and the S2 males show the least likelihood to playfully engage, and evade the most. Our previous work has also shown that whereas the subordinate rats learn fear conditioning by proxy from the dominant, the dominant does not learn from the subordinates^16^. Interestingly, males substantially diverge from females in their frequency of play behavior as they reach puberty^52^, leading to a general conclusion that play behavior is of lesser importance to female relationship management^51^.

### Differences in play styles in males versus females

In rats, play fighting involves one rat initiating contact to the nape followed by a defensive response by the recipient to avoid such a contact^28,55^. Defensive responses can include either evasion or facing defense. Facing defense includes either a standing retaliation (e.g. counter attack) or a partial or full rotation to a supine position^18,28,29,56^. In both males and females, these behaviors are mechanically the same but the frequency at which they occur differs between sexes and may have different functions.

Consistent with prior research in Long Evans and Norway rats^29,30,51–53^, we found that female Sprague Dawley rats differed from males not just in the frequency of play, with females initiating fewer nape contacts than males, but also in the likelihood of specific defensive response, with females evading more than males and engaging facing defense (e.g. counter attacks or rotations) less than males. It has been previously argued that the increase in frequency to evade when females are compared to males is in part due to the greater sensory sensitivity of females to nape approach^29^. Pellis^29^ postulated that this allows for more evasive responses due to the increased response time available for females. Also consistent with previous literature, the use of rotations onto supine and counterattacking are not as informed by social role in females as much as they are males, with male MNC (subordinate) roles being the most likely to rotate and HNC (dominant) males being the most likely to counterattack in response to a nape contact^29^.

We further show that asymmetry in nape contacts and the response to nape contacts during play sessions prior to fear conditioning by proxy can be used to predict socially acquired freezing to a cue in both male and female adult rats. In both sexes, demonstrators who were identified as HNC from play behavior transmitted highest levels of fear socially (or led their observer to acquire the most fear by-proxy); however, some important differences emerged. In males, rats that were most likely to respond to nape contacts with evasion displayed more socially learned fear behavior (LNC observers). In females, higher evasion rates were most likely to be observed in the HNC female who often displayed lower social fear learning. In addition, male rats that displayed the most countering behavior, often the HNC males, were more effective fear demonstrators, but the greatest countering behavior in female rats was observed in MNC animals. This could be due, in part, to fundamental differences in the responses to play initiation and response in males and females. Female HNC rats are most likely to respond to nape contact attempts with evasion whereas male HNC rats are most likely to respond to nape contact attempts with countering. LNC rats are most likely to evade when receiving a nape contact in males^29^.

This work highlights the importance of describing social relationships between observers and demonstrators in social learning paradigms, as well as emphasizes the need to conduct further work to examine how social behaviors and relationships differ between sexes.

### Social role and FCbP

As previously mentioned, females employ different types of behavior tactics from males during play, such as evasion which frequently occurs before a nape contact can be initiated^29^. This can influence the number of nape contacts initiated and received during a play session. Indeed, other investigations of female social role have involved other identifying rank by recording behaviors such as passing behavior in a burrow environment (e.g. which conspecific gets the “right of way” in a narrow tunnel) and resource monopolization investigations (e.g. which conspecific gets primary access to a desired resource)^20,22^. We argue that for the role classification presented above, the consistency of the findings in fear conditioning by proxy between males and females (with HNC rats being more effective fear demonstrators) suggests that there is a value to identifying social roles in females using one male typical dominance classification measure. In this framing, it is only the play behavior frequency and type of responses to nape contacts which seem to be unique to females. It has been suggested that in males, increased playful attention from the subordinate cage mates during short play sessions following periods of isolation may be an attempt to maintain a “friendly” relationship with the dominant animal^28^. Another theory for the value of play behavior is that play fighting allows young rats to rehearse fighting encounters that might happen in adulthood, in a battle for a mate or a resource, in a non-survival environment^57^. This theory is used to explain the difference in frequency of male and female play fighting, since virgin female rats aggressively compete for resources in the wild less often than males and also engage in less play fighting^29^. It is prudent to avoid assuming that female social behaviors should be attributed to have the same social significance as when they are performed by males, because males may be influenced by need for a different type of conflict resolution than females.

A limitation of our current classification of socially transmitted fear is that we only examined freezing at long-term memory. Without additional measures, such as ultrasonic vocalizations, social investigative behaviors during the task, other markers of fear during the CS such as vigilant scanning, or a more complete understanding of the real time olfactory communication between HNC and LNC observers or demonstrators, we cannot disambiguate whether the differential freezing from an HNC or LNC observer or demonstrator come from the HNC rats being more effective demonstrators or LNC rats being more effective observers. Still, our previous work offers some context. In a previous study by Jones and Monfils in 2016^16^, ultrasonic vocalizations (USVs) were recorded from male rats during fear conditioning, fear conditioning by proxy, and long term memory testing. We found that USVs were rarely emitted during the fear conditioning by proxy task but when vocalizations were produced, they significantly predicted freezing behavior of the observer during the long term memory test. Future studies will more comprehensively investigate the impact of USVs but they will need to be sufficiently powered such that there will be enough USV occurences in Fear Conditioning and Fear Conditioning by Proxy that meaningful correlations can be effectively interpreted.

In Jones and Monfils^16^, we found that more dominant demonstrators, either D demonstrators to S1 and S2 observers or S2 demonstrators to S1 observers, resulted in the greatest freezing by the observer. Given that there was strong kinship observed between dominant and subordinate 1 play partners in that study, and very little kinship observed at all between subordinate 2 play partners and dominant rats, but increased freezing behaviors were still observed when subordinate 2 rats observe dominant demonstrators, it appears that nape contacts in male rats were indicating something more than affiliative relationships in male rats^16^. These sub comparisons were not run in the current study, however, so future research should address other measures of fear communication, besides freezing, in order to better understand the methods that HNC demonstrators and/or LNC observers are employing to communicate fear during Fear Conditioning by Proxy.

### Is nape contact indicative of female dominance?

Interestingly, in the laboratory studies by Pellis and Pellis^58^, when the dominant male was removed from the cage, it was usually the low nape contacted animal that assumed the dominant role as opposed to the animal with the second highest nape contacts. This deepens the complexity of our understanding of the affiliative function of social play behaviors, suggesting that the most affiliative rat (as identified by the second highest number of nape contacts received and greatest number of nape contacts initiated) is more subordinate than the rat that is next in line for social dominance^58^. It follows that a rat that is least likely to become a dominant and independently have access to resources would be more playful and affiliative with the dominant in order to ensure a positive relationship that may lead to resource sharing^57^. Previous work in our lab with male rats has also demonstrated that the rat with the second most nape contacts (the true subordinate) readily learns fear conditioning from either the most (the dominant) or least (the second in line for dominance) nape contacted rat in the triad. Yet, the least nape contacted rat only learns from the dominant^16^. This seems to suggest the value placed on threat information is, at least in part, influenced by dominance status. The results of this experiment present an interesting extension of these findings.

In the course of our experiment, we tracked estrus, and found that there was a trend towards increased cornified cell frequency in HNC and LNC females compared to vaginal cell cytology shown in subordinate females (MNC), indicative of more days spent in mate-receptive status. This trend towards increased cornified cell frequency in HNC females likely correspond to the slightly increased (though not significant) estrus frequency. This is interesting given that in Adams and Boice^19^ female rats showed the greatest dominance like behavior when they were pregnant or lactating. It follows, then, that estrus cycle changes may influence dominance-like behavior and need to be further investigated to identify if play behavior is similarly influential or affected by estrus state. In future studies, it would also be valuable to continue tracking estrus cycle throughout fear conditioning in order to determine if fear experience influences estrus cycle, and if the stage of estrus phase that a female is conditioned in influences her fear expression in a long-term memory test^59^. Further investigation, involving consistent estrus cycle tracking being backed up by play behavior within the cage as well as a desired resource monopolization task to verify the most dominant female based on resource hoarding, is necessary to better understand how reliably estrus cycle can be utilized to determine dominance status of females.

We hesitate to assign nape contacts as dominance classification in females, though it appears that the relationship between females influences the value placed on social fear learning. Indeed, LNC observers learned fear conditioning readily from HNC demonstrators whereas HNC observers did not learn from LNC demonstrators. This suggests that a female’s social role influences the value placed on her demonstrated fear, as we see in males. This finding suggests a need to further investigate if female play behavior as a classifier for dominance results in unique outcomes in different social learning paradigms. In other words, we have shown that one measure of male typical dominance assignment results in similar outcomes for females in social fear learning as males.

### General discussion and conclusions

The present study aimed to evaluate the power of nape contacts received as a predictor of female expression of socially acquired fear. This method of social role quantification not only significantly predicted socially acquired fear in a way that was consistent with the findings of Jones and Monfils^16^ in males, it also had implications for play behavior in females. Though we are hesitant to claim that female dominance hierarchies exist in a male-congruent way, this study suggests that social rank exists in female rats, as represented by their consistent roles in play behavior and prioritization of nape determined rank in the expression of learned fear. Further research should investigate the value of other measures used to assign male dominance hierarchies to predict female social and fear expression behaviors. Additionally, the influence of play behavior role on desired resource access and hormonal environment (such as estrus characterization) should be further investigated.

### Supplementary Information

Supplementary Information.

## Data Availability

All data and R-codes are available at the Monfils Memory Lab repository, hosted by the University of Texas at Austin: https://dataverse.tdl.org/dataset.xhtml?persistentId=doi:10.18738/T8/PXOYJN.

## References

[1] John ER, Chesler P, Bartlett F, Victor I (1968). Observation learning in cats. Science.

[2] Hygge S, Ohman A (1978). Modeling processes in the acquisition of fears: Vicarious electrodermal conditioning to fear-relevant stimuli. J. Pers. Soc. Psychol..

[3] Mineka S, Cook M (1993). Mechanisms involved in the observational conditioning of fear. J. Exp. Psychol. Gen..

[4] Kavaliers M, Colwell DD, Choleris E (2005). Kinship, familiarity and social status modulate social learning about “micropredators” (biting flies) in deer mice. Behav. Ecol. Sociobiol..

[5] Olsson A, Nearing KI, Phelps EA (2007). Learning fears by observing others: The neural systems of social fear transmission. Soc. Cogn. Affect. Neurosci..

[6] Gonzalez-Liencres C, Juckel G, Tas C, Friebe A, Brüne M (2014). Emotional contagion in mice: The role of familiarity. Behav. Brain Res..

[7] Monfils MH, Agee LA (2019). Insights from social transmission of information in rodents. Genes Brain Behav..

[8] Agee LA, Jones CE, Monfils M-H (2019). Differing effects of familiarity/kinship in the social transmission of fear associations and food preferences in rats. Anim. Cogn..

[9] Jones CE, Riha PD, Gore AC, Monfils M-H (2014). Social transmission of Pavlovian fear: Fear-conditioning by-proxy in related female rats. Anim. Cogn..

[10] Kiyokawa Y, Honda A, Takeuchi Y, Mori Y (2014). A familiar conspecific is more effective than an unfamiliar conspecific for social buffering of conditioned fear responses in male rats. Behav. Brain Res..

[11] Pisansky MT, Hanson LR, Gottesman II, Gewirtz JC (2017). Oxytocin enhances observational fear in mice. Nat. Commun..

[12] Liévin-Bazin A, Pineaux M, Clerc O, Gahr M, von Bayern AMP, Bovet D (2018). Emotional responses to conspecific distress calls are modulated by affiliation in cockatiels (*Nymphicus hollandicus*). PLoS ONE.

[13] Chen Q, Panksepp JB, Lahvis GP (2009). Empathy is moderated by genetic background in mice. PLoS ONE.

[14] Jeon D (2010). Observational fear learning involves affective pain system and Cav1.2 Ca2+ channels in ACC. Nat. Neurosci..

[15] Hirota Y (2020). Oxytocin receptor antagonist reverses the blunting effect of pair bonding on fear learning in monogamous prairie voles. Horm. Behav..

[16] Jones CE, Monfils MH (2016). Dominance status predicts social fear transmission in laboratory rats. Anim. Cogn..

[17] Kendal R (2015). Chimpanzees copy dominant and knowledgeable individuals: Implications for cultural diversity. Evol. Hum. Behav..

[18] Pellis SM, Pellis VC (1992). Juvenilized play fighting in subordinate male rats. Aggress. Behav..

[19] Adams N, Boice R (1983). A longitudinal study of dominance in an outdoor colony of domestic rats. J. Comp. Psychol..

[20] Ziporyn T, McClintock MK (1991). Passing as an indicator of social dominance among female wild and domestic Norway rats. Behaviour.

[21] Clarke FM, Faulkes CG (1997). Dominance and queen succession in captive colonies of the eusocial naked mole-rat, *Heterocephalus glaber*. Proc. R. Soc. B.

[22] Parent CI, Del Corpo A, Cameron NM, Meaney MJ (2013). Maternal care associates with play dominance rank among adult female rats. Dev. Psychobiol..

[23] Lee CT, Naranjo JN (1974). The effects of castration and androgen on the social dominance of BALB/cJ male mice. Physiol. Psychol..

[24] Benton D, Dalrymplealford JC, Brain PF (1980). Comparisons of measures of dominance in the laboratory mouse. Anim. Behav..

[25] So N, Franks B, Lim S, Curley JP (2015). A social network approach reveals associations between mouse social dominance and brain gene expression. PLoS One.

[26] Williamson CM, Lee W, Curley JP (2016). Temporal dynamics of social hierarchy formation and maintenance in male mice. Anim. Behav..

[27] Williamson CM (2019). Social hierarchy position in female mice is associated with plasma corticosterone levels and hypothalamic gene expression. Sci. Rep..

[28] Pellis SM, Pellis VC (1996). On knowing it’s only play: The role of play signals in play fighting. Aggress. Viol. Behav..

[29] Pellis SM, Field EF, Smith LK, Pellis VC (1997). Multiple differences in the play fighting of male and female rats. Implications for the causes and functions of play. Neurosci. Biobehav. Rev..

[30] Marquardt AE, VanRyzin JW, Fuquen RW, McCarthy MM (2023). Social play experience in juvenile rats is indispensable for appropriate socio-sexual behavior in adulthood in males but not females. Front. Behav. Neurosci..

[31] Smith LK, Field EF, Forgie ML, Pellis SM (1996). Dominance and age-related changes in the play fighting of intact and post-weaning castrated male rats (*Rattus norvegicus*). Aggress. Behav..

[32] Kamitakahara H, Monfils MH, Forgie ML, Kolb B, Pellis SM (2007). The modulation of play fighting in rats: Role of the motor cortex. Behav. Neuro..

[33] Bell HC, McCaffrey DR, Forgie ML, Kolb B, Pellis SM (2009). The role of the medial prefrontal cortex in the play fighting of rats. Behav. Neurosci..

[34] Himmler BT, Pellis VC, Pellis SM (2013). Peering into the dynamics of social interactions: Measuring play fighting in rats. J. Vis. Exp..

[35] Schweinfurth MK (2017). Do female Norway rats form social bonds?. Behav. Ecol. Sociobiol..

[36] Schweinfurth MK, Stieger B, Taborsky M (2017). Experimental evidence for reciprocity in allogrooming among wild-type Norway rats. Sci. Rep..

[37] Fang J, Clemens LG (1999). Contextual determinants of female-female mounting in laboratory rats. Anim. Behav..

[38] Blanchard DC, Fukunaga-Stinson C, Takahashi LK, Flannelly KJ, Blanchard RJ (1984). Dominance and aggression in social groups of male and female rats. Behav. Process..

[39] Redecker TM, Kisko TM, Schwarting RKW, Wöhr M (2019). Effects of *Cacna1c* haploinsufficiency on social interaction behavior and 50-kHz ultrasonic vocalizations in adult female rats. Behav. Brain Res..

[40] Calhoun JB (1963). The Ecology and Sociology of the Norway Rat.

[41] Bruchey AK, Jones CE, Monfils M-H (2010). Fear conditioning by-proxy: Social transmission of fear during memory retrieval. Behav. Brain Res..

[42] Goldman JM, Murr AS, Cooper RL (2007). The rodent estrous cycle: Characterization of vaginal cytology and its utility in toxicological studies. Dev. Reprod. Toxicol..

[43] Westwood FR (2008). The female rat reproductive cycle: A practical histological guide to staging. Toxicol. Pathol..

[44] Venables WN, Ripley BD (2002). Modern applied statistics with S.

[45] R Core Team. R: A language and environment for statistical computing. R Foundation for Statistical Computing, Vienna, Austria. https://www.R-project.org/ (2020).

[46] Fox J, Hong J (2009). Effect displays in R for multinomial and proportional-odds logit models: Extensions to the effects package. J. Stat. Softw..

[47] Fox J, Weisberg S (2018). Visualizing fit and lack of fit in complex regression models with predictor effect plots and partial residuals. J. Stat. Softw..

[48] Fox J, Weisberg S (2019). An R Companion to Applied Regression.

[49] Kleiber C, Zeileis A (2008). Applied Econometrics with R.

[50] Bates D, Mächler M, Bolker BM, Walker SC (2015). Fitting linear mixed-effects models using lme4. J. Stat. Softw..

[51] Pellis SM, Pellis VC (1990). Differential rates of attack, defense, and counterattack during the developmental decrease in play fighting by male and female rats. Dev. Psychobiol..

[52] Meaney MJ, Stewart J (1981). A descriptive study of social development in the rat (*Rattus norvegicus*). Anim. Behav..

[53] Meaney MJ, Stewart J (1981). Neonatal-androgens influence the social play of prepubescent rats. Horm. Behav..

[54] Byers SL, Wiles MV, Dunn SL, Taft RA (2012). Mouse estrous cycle identification tool and images. PLoS One.

[55] Siviy SM, Panksepp J (1987). Sensory modulation of juvenile play in rats. Dev. Psychobiol..

[56] Whishaw IQ, Pellis SM, Pellis VC (1992). A behavioral study of the contributions of cells and fibers of passage in the red nucleus of the rat to postural righting, skilled movements, and learning. Behav. Brain Res..

[57] Smith PK (1982). Does play matter? Functional and evolutionary aspects of animal and human play. Behav. Brain Sci..

[58] Pellis SM, Pellis VC (1993). Some subordinates are more equal than others: Play fighting amongst adult subordinate male rats. Aggress. Behav..

[59] Blair RS, Acca GM, Tsao B, Stevens N, Maren S, Nagaya N (2022). Estrous cycle contributes to state-dependent contextual fear in female rats. Psychoneuroendocrinology.

