# Supplementary material for: Conspecific interactions predict social transmission of fear in female rats
Source: Sci Rep. 2024 Apr 2;14:7804. doi: 10.1038/s41598-024-58258-6 (PMC10987648; doi:10.1038/s41598-024-58258-6)
Supplement: Supplementary file 1 — Supplementary Information. [file 41598_2024_58258_MOESM1_ESM.docx]

**Conspecific interactions predict social transmission of fear in female rats**

Sydney-O. Seese*, Carolyn-E. Tinsley*, Grace Wulffraat, J. Gregory Hixon,
Marie-H. Monfils

* These two authors contributed equally to this work and share first authorship

**Supplementary Figures** p2-p9

**Contact Information:**

Marie H. Monfils

[marie.monfils@utexas.edu](mailto:marie.monfils@utexas.edu)

The University of Texas at Austin

Department of Psychology

108 E. Dean Keeton Stop A8000

Austin, TX 78712-1043

**Social Determination of Rank - Social Interactions**

In some cages, social role assignment, as determined by nape contacts received and response to nape contacts, was unclear or assigned incorrectly. Cages 7 and 12 demonstrated unclear assignments, having unclear LNC and MNC roles based on nape contacts initiated and received. In addition, Cage 1 was removed from consideration for all analyses, as the MNC role was fear conditioned instead of the HNC or LNC role. Please note, Cage 12 has 2 points on the LNC bin due to the subordinate ranking uncertainty. A potential future direction is to have a larger group of female cages and evaluate a greater number of inconsistent cages for individual characteristics that make the social ranking unique (such as indistinct estrus phases).


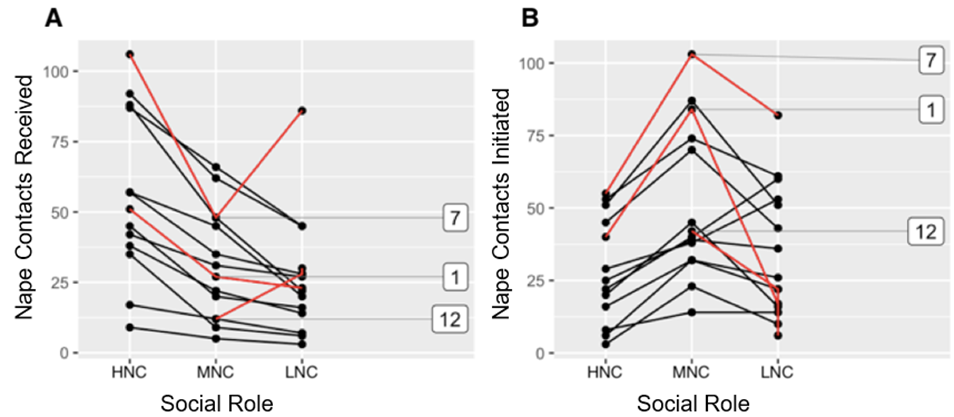


**Supplementary Figure S1:** Cages 1, 7, and 12 were removed from play behavior analysis due to unclear social roles. Cages 1, 7 and 12 were removed from Fear Conditioning by Proxy analysis due to inconsistent demonstrator/observer assignment. **A:** number of nape contacts received by each member of the cage triad. **B:** number of nape contacts initiated by each member of the cage triad.

**Effect of Social Rank Cue on Conditioned Freezing**

Cue had an effect on freezing behavior during long term memory testing (LTM) in the fear conditioned by proxy (FCbP) Low Nape Contacted (LNC) males. Mean freezing response was significantly increased in LNC males from Cue 1 to Cue 2 in the LTM test (t(15.983) = -2.196, p = 0.043). Additionally, during the LTM test, male FCbP LNC rats showed greater freezing response during the second FCbP cue than female FCbP LNC (t(10.43) = 2.31, p = 0.043). No other between cue comparisons were significant in LNC or HNC females or HNC males of either the direct fear conditioned or fear conditioned by proxy condition, nor were any other between cue comparisons significant between males and females of either social role or fear conditioning group.

Importantly, the male data presented below and in the main paper are selected for male demonstrators and observers that were only LNC and HNC. These males either went through fear conditioning (FC), or were fear conditioned by proxy (FCbP). The remaining MNC males are only presented if they were in the no fear conditioning group (NFC). As such, even though the subject pool was larger for males (with MNC males going through FC and FCbP, as well as HNC and LNC males going through NFC), we are providing a reduced subject number for the sake of comparison with the female group, including only observers (fear conditioned by proxy) that were LNC and HNC as well as demonstrators (directly fear conditioned) that were LNC and HNC, with MNC males as the no fear conditioning group.

**Supplementary Figure S2 (A):** CS freezing at LTM in females


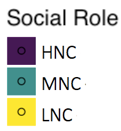

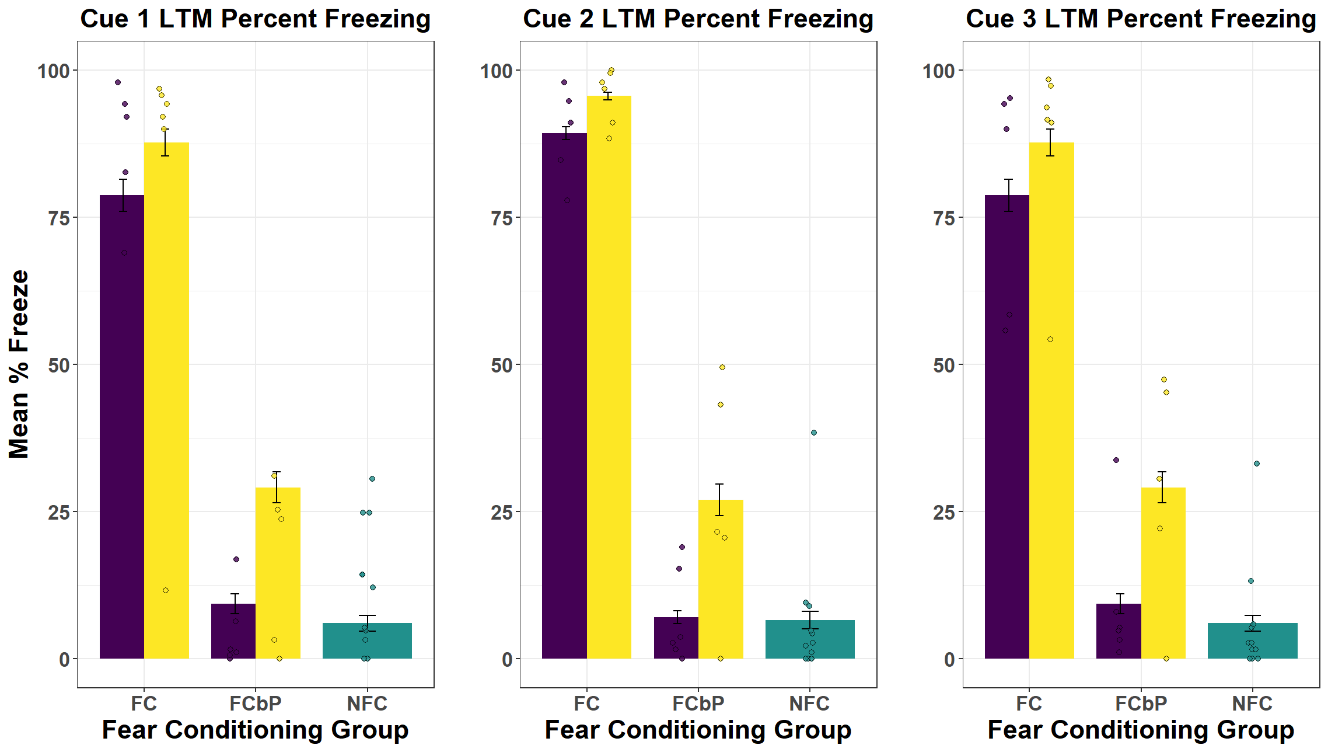


**Supplementary Figure S2 (B):** CS Freezing at LTM in males.


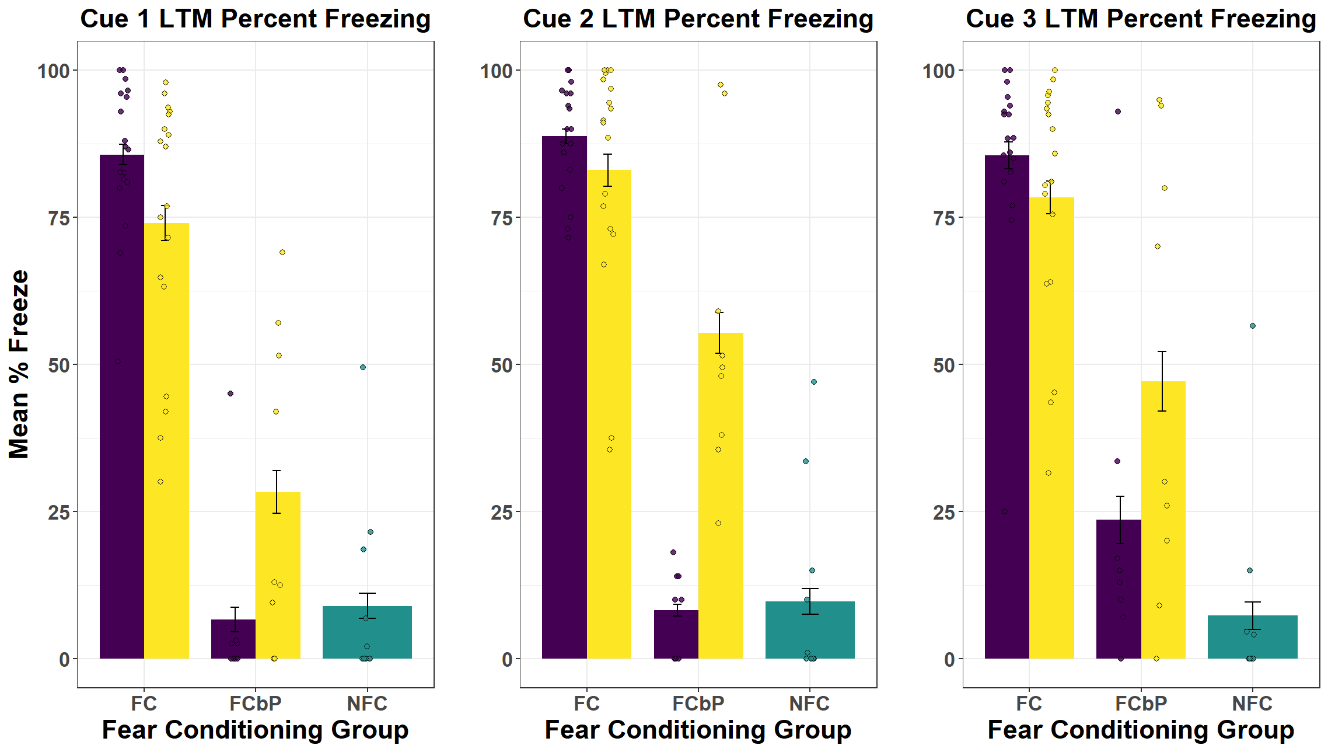


**Supplementary Figure S2 (A):** Mean CS freezing by all females based on social role and number of cue presentations (three conditioned stimuli were presented total during the LTM test).

**Supplementary Figure S2 (B):** Mean CS freezing by a subset of all males tested (including only demonstrators and observers who were LNC or HNC and only MNC NFC subjects) based on social rank and number of cue presentations.

**Effect of Demonstrator Freezing During FCbP on Observer Freezing at LTM**

A linear model was built to evaluate the association between demonstrator freezing and observer freezing when the demonstrator is freezing at FCbP and when the observer is freezing at LTM. Demonstrator freezing at FCbP does not significantly predict observer freezing at LTM (F(1,9)= 0.889,p=0.370). This is consistent with previous findings [Bruchey et al., 2010; Agee, Jones & Monfils, 2018] that demonstrator freezing at FCbP does not significantly predict observer freezing. Also of note, during FCbP, observers exhibited very little freezing, froze significantly less than demonstrators (F(1,19) = 79.769, p = 3.146e-08), and showed no significant differences in FCbP mean percent freezing between social rank (F(1,19) = 1.511, p = 0.234). Similarly, there were no significant differences in percent freezing between rats of different social ranks at every FC cue during direct fear conditioning (Cue 1: t(8.268) = -1.047, p = 0.325; Cue 2: t(8.929) = 0.488, p = 0.637; Cue 3: t(8.738) = 0.146, p = 0.887). HNC and LNC demonstrators showing no significant differences in percent freezing during direct fear conditioning (FC) or the FCbP task, suggesting that the enduring effects of observing HNC vs LNC demonstrators for FCbP observers are not as a result of different levels of freezing during the FCbP training session.

It is possible that the rats transmitted information during the FCbP session via producing ultrasonic vocalizations. Indeed, in a previous study, we observed that a subset of males produced ultrasonic vocalizations during the Fear Conditioning by Proxy task (Jones and Monfils, 2016). We found that when present, USVs significantly predicted the effectiveness of social transmission of fear, but that they were rarely emitted overall in the task (only 10 rats vocalized during FCbP). It is also unclear whether the vocalizations emitted during the FCbP test were performed by the demonstrator, the observer, or both. It worth noting that in long-term memory test, only directly fear conditioned rats vocalized [Jones & Monfils, 2016].

**Supplementary Figure S3:** Demonstrator Freezing at FCbP and Observer Freezing at LTM in females


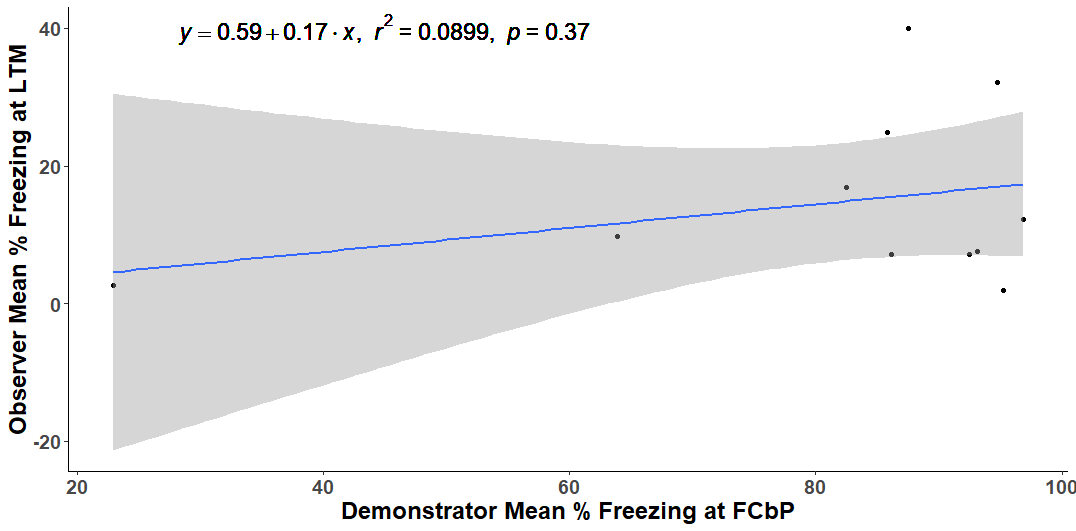


**Supplementary Figure S3:** Mean freezing of each demonstrator across all cues in the FCbP task plotted against mean freezing of each observer across all cues in the LTM task. Demonstrator freezing behavior during the FCbP task did not significantly predict observer freezing during the LTM task (R^2 and p values are presented on the graph).

**Supplementary Figure S4:** Demonstrator and Observer Freezing During FCbP Task


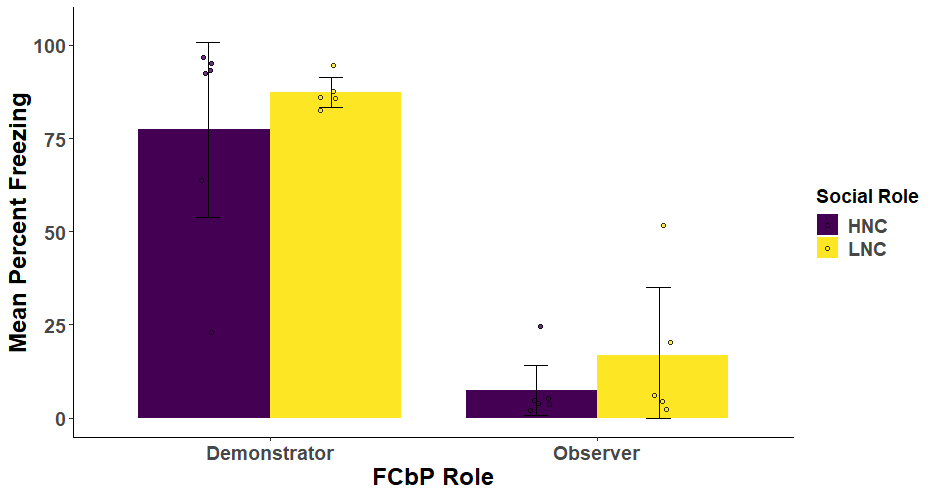


**Supplementary Figure S4:** Mean freezing of demonstrators and observers by social role. There was no significant difference in freezing behavior between social roles during the FCbP task, though there was significantly less freezing in observers of both ranks than in demonstrators of both ranks.

**Supplementary Figure S5:** Freezing between Social Ranks During Direct Fear Conditioning


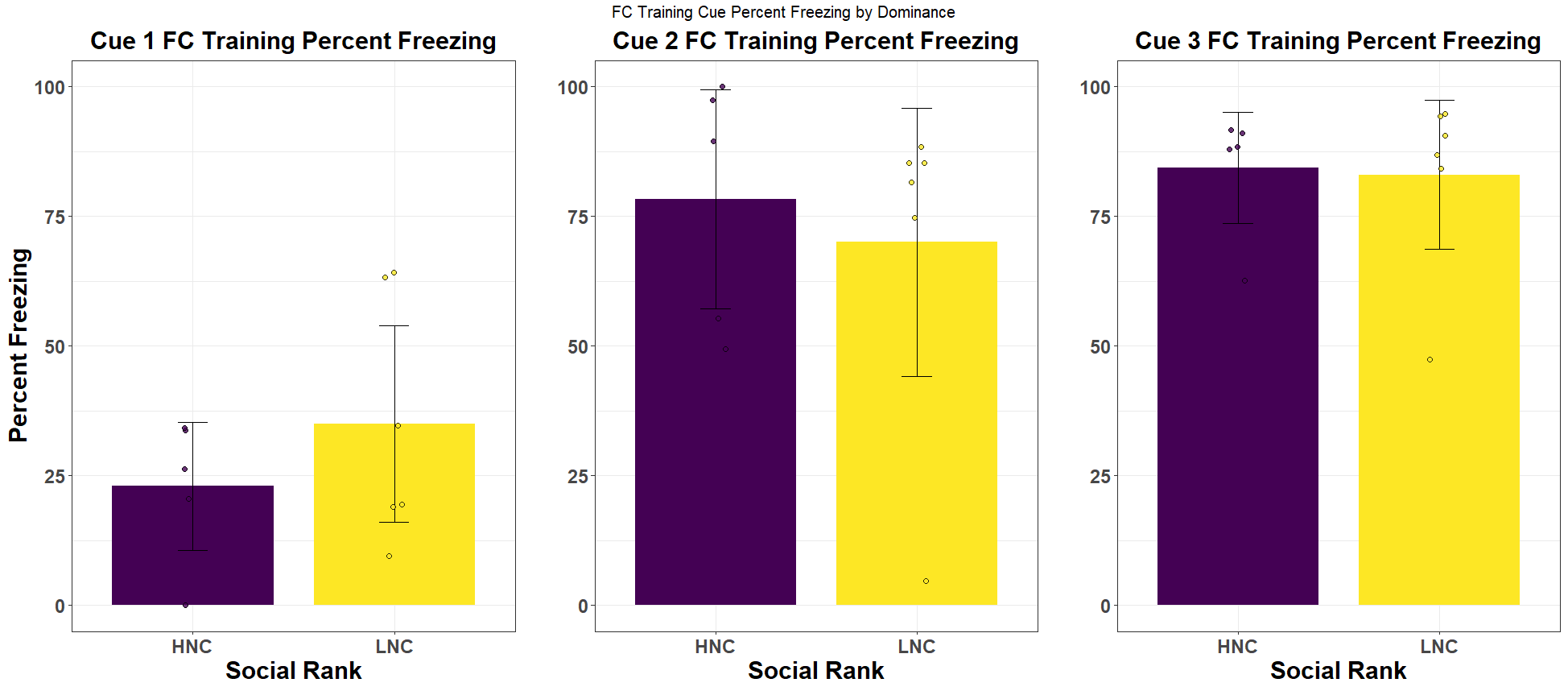


**Supplementary Figure S5:** There were no significant differences in percent freezing during direct fear conditioning between HNC and LNC female rats. This is consistent with our finding presented in Supplemental Figure 4, that there were no significant differences in mean percent freezing between HNC and LNC female demonstrators during the FCbP demonstration period.

**Effect of Sex on Nape Contacts Initiated and Received**

The literature has long supported that males have a higher frequency play behavior than females once puberty is reached [Meaney & Stewart 1981]. This was demonstrated in our data as well, with males showing significantly more nape contacts that were received during play and a trend towards more nape contacts initiated during play [initiated: $\chi^{2}\left( 1 \right)= 3.17, p=0.075$, received: $\chi^{2}\left( 1 \right)= 13.83, p=2.01e-04$]. These findings are plotted below.


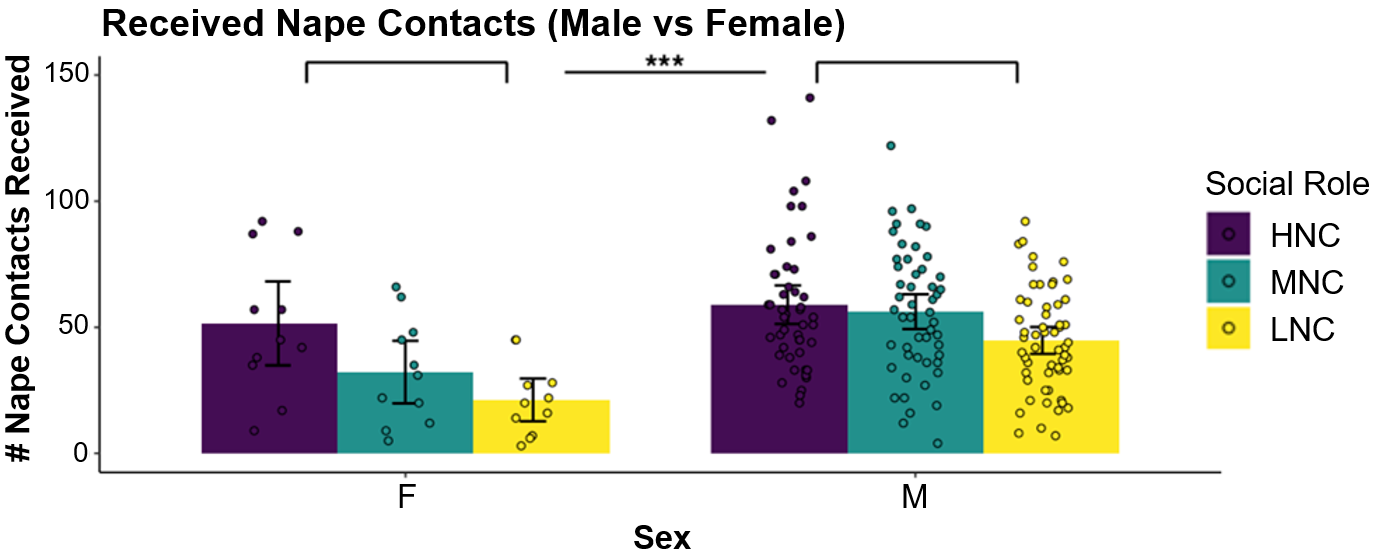


**
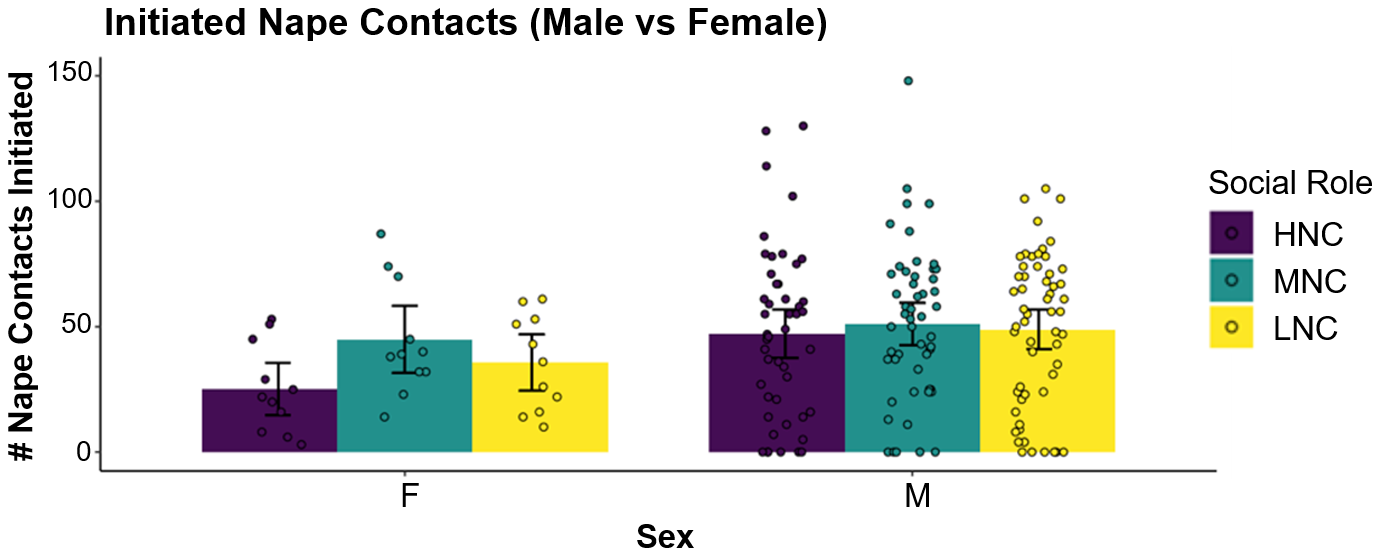
**

**Supplementary Figure S6 (Top):** Number of nape contacts received in male and female cages based on social rank during play behavior (as assigned by HNC, MNC, LNC). Males had significantly more nape contacts received than females, perhaps indicating greater play frequency.

**Supplementary Figure S6 (Bottom):** Number of nape contacts initiated in male and female cages based on social rank during play behavior (as assigned by HNC, MNC, LNC). Males showed a trend towards significantly more nape contacts initiated than females.

**Relationship Between Nape Contacts and Vaginal Cell Cytology**

We found no significant relationships between social role during play the frequency of proestrus, estrus, or diestrus ($proestrus: \chi^{2}\left( 2 \right)=0.16,p=0.92, estrus: \chi^{2}\left( 2 \right)=2.43,p=0.30, diestrus: \chi^{2}\left( 2 \right)=1.61,p=0.45$) see Figure S4. However, we did find that HNC and LNC females showed greater cornified estrus cell frequency (measured by 2 or more days of cornified cells observed in the 11 days of smear tracking) than MNC females. Though this difference was non-significant, it presented a moderately strong effect size (MNC role compared to LNC/HNC roles: ß = -1.74, p = 0.15; Effect size HNC vs. MNC: Cohen’s D = 0.66, LNC vs. MNC: Cohen’s D = 0.66) see Figure S5.


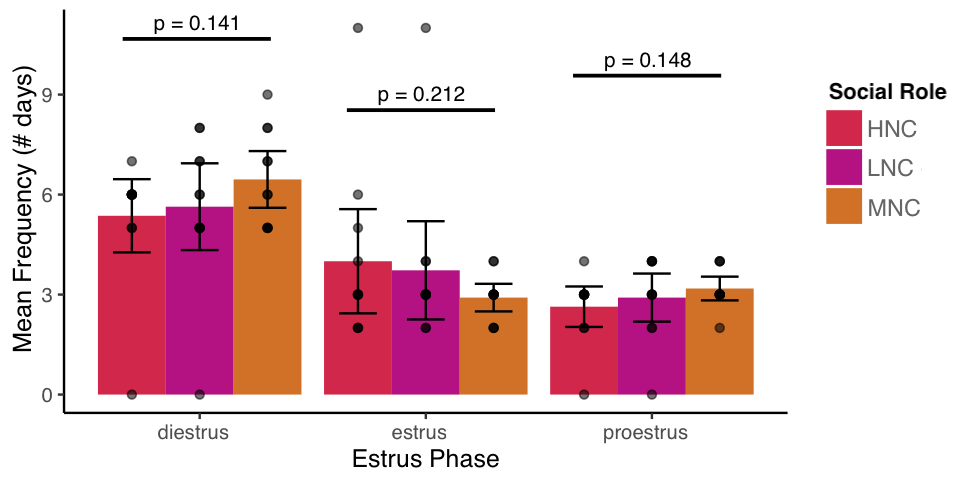


**Supplementary Figure S7: Mean number of days in diestrus, estrus, and proestrus across nape contact assignments.** 95% confidence intervals for each group mean are displayed as error bars, black points reflect individual frequencies of estrus phase. HNC and LNC females do not demonstrate significant differences in sexually receptive vaginal states compared to MNC females. But, there appears to be a slight trend towards less frequent diestrus and proestrus in HNC females, as well as a very slight trend towards more frequent estrus, compared to MNC females. The large individual variability of estrus frequency in across social roles in females appears to be blunting the effect of social role on estrus state.


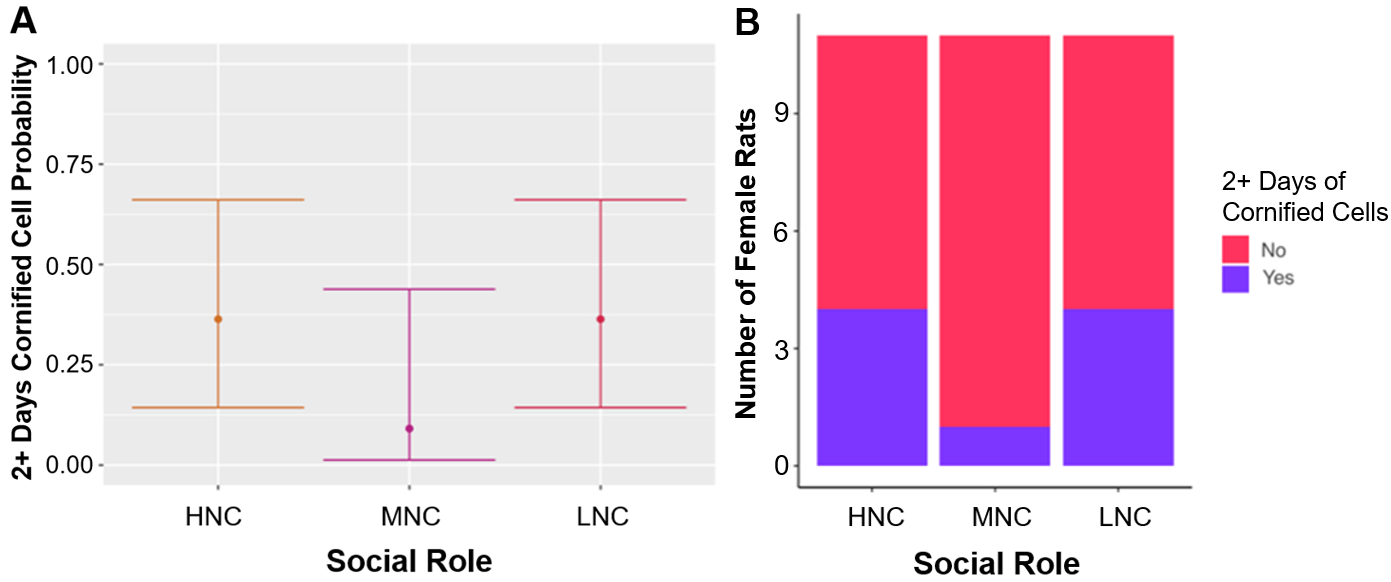


**Supplementary Figure S8:**

**Panel A: Logistic regression predicted frequency 2 or more days of cornified estrus vaginal cytology across rank.** HNC and LNC females are predicted to have longer cornified epithelia cell presence in vaginal cellular environment compared to MNC females.

**Panel B: Number of female rats with 2 or more days of cornified cell presence.** HNC and LNC females show longer period of cornified epithelia cell type present in vaginal cellular environment compared to MNC females.

**Methods for Scoring Play Behaviors**

| **Social Behavior** | **Description** |
| --- | --- |
| **Offensive** |  |
| Nape contact | snout of one rat is within 1 cm of the nape |
|  | of another; not aggressive grooming |
|  |  |
|  |  |
| **Defensive** |  |
| Evasion | retreat and flee; movement directed away from |
|  | the approacher |
|  |  |
| Counter attack | successful launch of an attack at the initiating rat; |
|  | includes upright defensive posture |
|  |  |
| Full rorate | on the back posture; access to nape is blocked by |
|  | target rotating fully to expose the ventral surface |
|  |  |
| Half rotate | target rotates laterally to block nape access but |
|  | hind feet remain planted |
|  |  |

**Supplementary Table S1. Social Behaviors Scored During Social Interaction Tests**

Descriptions of offensive and defensive behaviors tallied across three social interaction sessions.
